# Childhood physical abnormalities following paternal exposure to sulfur mustard gas in Iran: a case-control study

**DOI:** 10.1186/1752-1505-4-13

**Published:** 2010-07-14

**Authors:** Hassan Abolghasemi, Mohammad H Radfar, Mehdi Rambod, Parvin Salehi, Hossein Ghofrani, Mohammad R Soroush, Farahnaz Falahaty, Yousef Tavakolifar, Ali Sadaghianifar, Seyyed M Khademolhosseini, Zohreh Kavehmanesh, Michel Joffres, Frederick M Burkle, Edward J Mills

**Affiliations:** 1 Research Center for Chemical Injuries, Baqiyatollah Medical Sciences University, Vanak Square, Tehran, Iran; 2 Urology & Nephrology Research Center, Shahid Beheshti University of Medical Sciences 9th Boostan Street, Pasdaran Avenue, Tehran, Iran; 3 Research Institute for Endocrine Sciences, Shahid Beheshti University of Medical Sciences, Velenjak, Tehran, Iran; 4 Janbazan Medical and Engineering Research Center (JMERC), Chemical Warfare Victims Research Unit, Velenjak, Tehran, Iran; 5 Faculty of Health Sciences, Simon Fraser University, Burnaby, Canada; 6 Harvard Humanitarian Initiative, Harvard School of Public Health, Harvard University, Boston, USA; 7 Interdisciplinary School of Health Sciences, Faculty of Health Sciences, University of Ottawa, Ottawa, Canada

## Abstract

**Background:**

Mustard gas, a known chemical weapon, was used during the Iran-Iraq war of 1980-1988. We aimed to determine if exposure to mustard gas among men was significantly associated with abnormalities and disorders among progenies.

**Methods:**

Using a case-control design, we identified all progenies of Sardasht men (exposed group, n = 498), who were born at least nine months after the exposure, compared to age-matched controls in Rabat, a nearby city (non-exposed group, n = 689). We conducted a thorough medical history, physical examination, and appropriate paraclinical studies to detect any physical abnormality and/or disorder. Given the presence of correlated data, we applied Generalized Estimating Equation (GEE) multivariable models to determine associations.

**Results:**

The overall frequency of detected physical abnormalities and disorders was significantly higher in the exposed group (19% vs. 11%, Odds Ratio [OR] 1.93, 95% Confidence Interval [CI], 1.37-2.72, P = 0.0002). This was consistent across sexes. Congenital anomalies (OR 3.54, 95% CI, 1.58-7.93, P = 0.002) and asthma (OR, 3.12, 95% CI, 1.43-6.80, P = 0.004) were most commonly associated with exposure. No single abnormality was associated with paternal exposure to mustard gas.

**Conclusion:**

Our study demonstrates a generational effect of exposure to mustard gas. The lasting effects of mustard gas exposure in parents effects fertility and may impact child health and development in the long-term.

## Introduction

Sulfur mustard gas [bis(2-chloroethyl)sulfide], first synthesized in early 1800 s, has been used in several major wars, and is a common chemical warfare agent [1]. Iraqi forces used it against civilian populations during the 1980-1988 Iran-Iraq war [2,3].

Although mustard gas can have severe systemic effects on humans [1], it is best known as a skin vesicant. In a series of approximately 34,000 Iranian patients exposed to mustard gas, the lungs, eye, and skin were the most common sites of injury, in order of the greatest prevalence [4,5].

Beside its acute effects, mustard gas has a number of known long-term effects on various body organs such as lung, stomach, bone marrow, and gonads [6-11]. In addition, sulfur mustard has been shown to influence the reproductive function in both animals and human being [9,12-14]. It is a potent carcinogen and mutagen [15]. However, there is little information about parental exposure to sulfur mustard and congenital anomalies in the offspring.

We aimed to estimate the frequency of physical abnormalities and disorders among the progenies of men in a major urban setting (Sardasht City) exposed to sulfur mustard gas. We aimed to compare the prevalence of observed abnormalities and disorders with those of a non-exposed population.

## Methods

### Subjects and setting

We began this study in 2004, about 17 years after the chemical attack in Sardasht. We followed children up to March 2009. Sardasht is a western Iranian city that was chemically bombarded with sulfur mustard gas (HD) by Iraqi forces on June 28, 1987. Documents maintained by military and civil authorities in Sardasht region confirm that approximately 8025 inhabitants were exposed to mustard gas and approximately 4500 people received medical treatment. Of these, records confirming early clinical manifestations of exposure and subsequent medical complications were available for 735 male survivors [9].

All Sardasht men that had a confirmed history of mustard gas exposure were selected and their progenies, who were born at least nine months after the exposure were considered as the exposed group. However, children whose both parents had confirmed history of exposure to mustard gas were not included in this study.

Since Iraqi forces attacked Iranian soldiers and civilians with various chemical agents several times, a special commission was assigned by Veteran Affairs Organization to confirm the chemical exposure and to determine the severity of injury. This commission consisted of a pulmonologist, a dermatologist, a neurologist, and an ophthalmologist that based their decision on hospital documents, mustard gas exposure stigmata, and clinical signs and symptoms of chronic complications of exposure, and appropriate paraclinical studies as required for diagnostic confirmation. All Sardasht men studied had official confirmation of chemical exposure from this commission. These patients were contacted using the Veteran Affairs Organization's database and by placing signposts and billboards across the city.

### Control population

Rabat is a nearby city that has ethnic, cultural, and geographical characteristics similar to Sardasht but was not exposed to chemical attack. Rabat is 20 Km from Sardasht, and according to governmental documents, no proven traces of chemical agents were found in the Rabat region. There is no difference in access to medical care between the two regions. In this study, 164 couples from Sardasht were frequency matched by age with 136 couples from an existing cohort from Rabat. The initial groups were larger due to differences in the age distribution, we used a random number table to remove randomly several cases and controls to obtain a similar distribution of individuals in each age group.

### Data Collection

We developed an interview sheet and a checklist as data-gathering tools. Our data collection included demographic data of all chemically injured males and their spouses in the exposed (Sardasht) group, age, occupation, educational level, date of marriage; close relative marriage, and any history of chemical injury. The same data was also gathered from the non-exposed (Rabat) group.

Trained general practitioners (GPs) evaluated medical histories from all progenies born at least nine months after the date of the chemical exposure in both exposed and non-exposed groups. In addition, the GPs performed a complete physical examination. If any physical abnormality or special disorder was suspected, the progeny was referred to a pediatrician to confirm the diagnosis. If the pediatrician considered additional diagnostic tools necessary for confirmation, paraclinical studies (e.g. echocardiography, spirometry, various imaging techniques) were performed.

Based on these examinations and confirmatory paraclinical studies, various abnormalities were identified and recorded. All abnormality entities were coded based on International Classification of Diseases, revision 10 (ICD-10) and/or disorders were classified as follows: diseases of the blood; endocrine, nutritional and metabolic diseases; mental and behavioral disorders; diseases of the nervous system and epilepsy; diseases of the eye, adnexa, ear and mastoid process; diseases of the circulatory system; diseases of the respiratory system; diseases of the digestive system; diseases of the musculoskeletal system; diseases of the genitourinary system; and congenital malformations. Whenever any disagreement occurred between the diagnostic opinion of GPs and the pediatrician, the pediatric diagnosis was accepted. In addition, if a progeny had a history of physical abnormality and disorder based on medical records but had normal physical exam at the time of the present study, due to previous medical treatment or surgical management, they were included as an event. Physical abnormalities or disorders were defined as any abnormality that can be detected by medical history, physical exam, or paraclinical studies.

Our sample size estimation was based on 95% power, a two-sided alpha (0.01), and an expected difference of 10% with a continuity correction. We provide descriptive statistics on the populations as groups. To take into account the correlation between families, we used SAS GENMOD procedure (SAS 9.2 SAS, Gary, NC), assumed a binomial distribution, logit link function and an exchangeable correlation matrix type.. We provide point estimates as Odds Ratios (ORs) with 95% Confidence Intervals (CIs). We used 2-sided P-values.

## Results

Two hundred and eighty-three couples had official confirmation of exposure to Sulfur Mustard gas. Of these, in 193 couples only the male partner was chemically injured at least nine months before conception. Nineteen of these men were infertile (primary infertility in 12 men); of the seven men with secondary infertility five men had become infertile after the exposure; one was infertile before exposure and the data of the last one was not available. Ten were older than 65 years at exposure. Therefore, this study included 164 couples as designated exposed group. The non-exposed group, also, consisted of 136 age-matched couples. General characteristics of exposed and non-exposed groups are summarized in Table 1.

**Table 1:** General characteristics of exposed (Sardasht) and non-exposed (Rabat) couples at the time of study

	Exposed (Sardasht) N = 164	Non- exposed (Rabat) N = 136	P-Value
**Age (years)**			
**Males**	45.9 (8.3)	44.4 (8.1)	0.14
**Females**	40.6 (8.7)	49.3 (14.9)	0.0001
**Duration of marriage**	20.5 (8.0)	23.6 (8.8)	0.002
**Close relative marriage**	7.3% (N = 12)	14.7% (N = 20)	0.04
**Education level (female partner)**			
**Illiterate**	14.0% (N = 23)	86.8% (N = 118)	0.0001
**Elementary**	26.2% (N = 43)	10.3% (N = 14)	
**High School**	17.1% (N = 28)	0.7% (N = 1)	
**Some or more college**	42.7% (N = 70)	2.2% (N = 3)	
**Number of Children**			
**Males**	291 (58%)	350 (51%)	0.009
**Females**	207 (42%)	339 (49%)	
**Total**	498	689	
**Average number of children per father**	3	5	
**Average number of brothers**	1.7	1.5	0.01
**Average number of sisters**	1.6	1.7	0.6

Data are presented as mean standard deviation (SD) or proportion (%).

Of a total number of 498 progenies born at least nine months after chemical attack in the exposed group, 291 (58%) were males (male/female ratio: 1:1.40). In Rabat, 689 children were born at least nine months after the date of Sardasht bombardment; 350 (50%) were males (male/female ratio: 1:1.03). Children in the non-exposed group were slightly older than their counterparts in the exposed group (10.6 standard deviation (SD) 4.7 years and 9.0 SD 4.7 years, respectively, P = < 0.0001). In both groups, the minimum child's age was 0.1 year and maximum age 16 years.

Based on the medical history, physical examination, and paraclinical studies, a comparative listing of abnormalities was prepared between the exposed and non-exposed groups. This list and related ICD-10 coding is provided in Table 2. In addition, the numbers of progenies whose abnormality was confirmed by medical history but were partially or completely normal at the time of physical examination because of prior medical intervention were 32 (6.4%) and 20 (2.8%) in the exposed and non-exposed groups, respectively (*P *= 0.19).

**Table 2:** List of physical abnormalities and disorders and their ICD-10 coding found in the progenies of males exposed and non-exposed to mustard gas

Condition*	ICD Coding	Exposed (Sardasht) (n = 498)	Non-exposed (Rabat) (n = 689)
Endocrine, Nutritional and Metabolic Diseases	**E Group**		
Hyperthyroidism	E 05	1	0
Addison's disease	E 27.1	1	1
Total			
Mental and Behavioral Disorders	**F Group**		
Mental retardation	F 70	4	0
Diseases of the Nervous System	**G Group**		
Epilepsy	G 40	0	1
Muscular dystrophy	G 71.0	0	1
Cerebral Palsy	G80	6	3
Total			
Diseases of the Eye, Adnexa, Ear and Mastoid Process	**H Group**		
Ambliopia	H 53.0	0	1
Strabismus	H 50	1	3
Ptosis	H 02.4	0	2
Congenital nistagmus	H 55	1	0
Hearing loss	H 90	1	1
Total		3	7
Diseases of the Circulatory System	**I/R Group**		
Rheumatic fever with heart involvement	I 01	1	1
Mitral valve prolapse	I 34.1	3	0
Arrhythmias	I 49	6	1
Cardiac murmur	R1	11	3
Total			
Diseases of the Respiratory System	**J Group**		
Asthma	J 45	20	9
Total		20	9
Diseases of the Digestive System	**K Group**		
Inguinal hernia	K 40	11	18
Umbilical hernia	K 42	1	0
Diaphragmatic hernia	K 44	1	0
Total		13	18
Diseases of the Musculoskeletal System and Connective Tissue	**M Group**	0	2
Torticollis	M 43.6	0	2
Total			
Diseases of the Genitourinary System	**N Group**		
Pyelonephritis	N 11	16	13
Nephrolithiasis	N 20.0	2	3
Total		18	16
Congenital Malformations	**Q group**		
Microcephaly	Q 02	1	1
Congenital cardiac disease	Q 21	1	1
Ventricular septal defect	Q 21.0	1	0
Tetralogy of Fallot	Q 21.3	1	0
Aortic stenosis	Q 23.0	1	0
Cleft palate	Q 35	0	1
Bicornate uterus	Q51.	1	0
Retractile/Undescended testis	Q 55.2	4	2
Congenital dislocation of hip	Q 65.0	1	1
Club foot	Q 66.0	4	0
Flat foot	Q 66.5	1	0
Congenital musculoskeletal deformities of head, face, spine and chest	Q 67	1	0
Pectus excavatum	Q 67.6	1	2
Phocomelia	Q73	2	0
Congenital malformation of knee	Q74.1	3	0
Total		23	8
Total		99	67

*Children may present with more than one condition

In the exposed and non-exposed groups, 120 (24%) and 52 (7.5%) patients were referred to the pediatrician for further investigation and confirmation of diagnosis. After this second-level assessment, the overall frequency of evaluated abnormalities and disorders in the exposed group was significantly higher than the non-exposed group (95 [19%] vs. 77 [11%]; 1.93, 1.37-2.72, *P *< 0.001).

According to the ICD-10 coding, 20 (4%) patients in the exposed group had respiratory diseases ("J" group) and 21 (4%) congenital malformations ("Q" group), respectively; while both these figures were 9 (1%) in the non-exposed group. Frequency of respiratory diseases (OR, 3.12, 95% CI, 1.43-6.80, *P *= 0.004) and congenital malformations (OR 3.54, 1.58-7.93, *P = *0.002) were significantly higher in the exposed group than in the non-exposed (See Table 2).

## Discussion

Our study found that the overall frequency of physical abnormalities is significantly associated with children whose fathers were exposed to mustard gas. Furthermore, there was a significant association between paternal exposure to mustard gas and both respiratory diseases and congenital malformations. Given the widespread use of mustard gas in wartime, the lasting effects may potentially last generations.

Animal and human studies indicate that paternal exposure to certain agents can result in developmental abnormalities in progenies [16]. Several studies demonstrated that exposure of male rats and mice to cyclophosphamide can lead to congenital malformations in progenies [17,18]. In humans, paternal exposure to various agents such as acrylamide, lead, and solvents result in an increase in congenital malformations [19-21]. A mechanism suggested for the effect of paternal preconception exposure is the occurrence of transmissible genetic changes or an epigenetic mechanism [22-24].

### Possible explanation of findings

Sulfur mustard is a cytotoxic agent with mutagenic and carcinogenic effects [15]. Its active intermediate, sulfonium ion, reacts rapidly with proteins and nucleic acids, alters chemical functional groups such as amines, carboxyls, phosphates, S-H, and O-H groups, and produces alkylation products. This process may result in cross-linking between adjacent strands of DNA, which has been shown to be extremely lethal to cells [6];

Confirmed effects of sulfur mustard gas on spermatogenesis may explain the observed overall increase in physical abnormalities among the progenies of chemical victims [2,12,25,26]. However, few studies, previously performed, were conclusive in determining a causal relationship. Pour-Jafari et al. [27] studied the rate of congenital malformations among progenies and their parents of Iranian victims before and after chemical warfare exposure and found that the rate of major malformations has increased from 33 per 1000 to 258 per 1000. Although they had used his cases as their own controls, and thus reduced selection bias, they did not adjust for the effect of parents' increasing age on malformation occurrence. Taher et al[28] claimed that the use of mustard gas in the Iran-Iraq conflict might have increased the number of cleft lip and cleft palate in children, however, they were not able to establish any causal effect between these two events, nor could they exclude the effects of other possible causes.

### Strengths & Limitations

Strengths of our study include its sample size and locally relevant controls. Sardasht is one of the rare instances in the world with a large population of mustard gas victims. Despite initial resistance to studying the effects of exposure, overtime, initial politico-ethical resistance has faded. No similar study has yet been performed with this population. Furthermore, presence of an unexposed population in a nearby city (Rabat) with characteristics similar to the exposed group provided a unique opportunity to further strengthen this analysis. An inherent limitation of studies like this, where the participants may consider probable benefits by over-reporting adverse outcomes, and also where a long time is passed from the date of the event under investigation, is the possibility of recall bias. We aimed to minimize these biases by an inclusive physical exam and appropriate paraclinical studies performed by GPs and further confirmation by a pediatrician. However, our exposed group included only the progenies of those exposed males who were present in the city at the time of study. Moreover, we do not know the number of men exposed that died or moved since exposure. We used clinical examination and paraclinical tests of live birth children to determine disorders and malformations. It is possible that kariotyping and other genetic studies could have revealed more problems [25,29]. It is also possible that miscarriages or abortions would have yielded differing effects. Finally, it is possible that our control population differs importantly from the exposed population that we have not recognized. This issue exists with any non-randomized comparison and we are unable to overcome this concern.

Our study found a significant association between exposure to mustard gas and common disorders and malformations. We did not find that any specific disorder or malformation was associated with exposure. We expected this as, with anencephaly, for example, there is a prevalence rate of about one in 1000 live births [1], thus, even with a doubling of risk rates, a much greater population would need to be studied to reveal strong association with chemical exposure. Considering the high number of chemical victims in Iran, this study may catalyze further comprehensive assessments with larger study populations. Using a *post hoc *sample size calculation, we find that our study had greater than 95% power to detect malformations and greater than 99% power to detect malformations/disorders.

We evaluated all clinical disorders and malformations, regardless of hypothesis driven associations with chemical exposure. Our reasoning for this is that the link between genetic disorders and resulting illnesses is not yet completely understood. So, for example, while rheumatic fever with congenital involvement may be most often associated with communicable disease genesis, we cannot rule out that parental chemical exposure may be associated with predispositions to certain illnesses [30].

### Interpretation

Our study found a significant association between overall frequency of physical abnormalities and disorders and paternal exposure to mustard gas. Given the considerable victims of mustard gas among Iranian civilians and military personnel, as well as civilians and military in other conflicts, the effects of war may have a lasting and important effect on generations to come.

#### Ethical statement

The Board of Research Ethics in the Janbazan Medical and Engineering Research Center (JMERC) and Shahed University approved this study. Informed written consent was obtained from all the parents and caregivers involved in this study.

## Competing interests

The authors declare that they have no competing interests.

## Authors' contributions

HA, MHR, MR, PS, HG, MRS, FF, YT, AS, SMK, ZK conceived the study. HA, MHR, MR, PS, HG, MRS, FF, YT, AS, SMK, ZK designed and conducted the data collection. HA, MHR, MR, PS, HG, MRS, FF, YT, AS, SMK, ZK, MJ, FMB, EJM conducted data analysis. HA, MHR, MR, PS, HG, MRS, FF, YT, AS, SMK, ZK, MJ, FMB, EJM wrote the drafts of the manuscript and approved the final submitted version.
